# Numerical Simulation and Experimental Study on the TIG (A-TIG) Welding of Dissimilar Magnesium Alloys

**DOI:** 10.3390/ma15144922

**Published:** 2022-07-15

**Authors:** Bo Qin, Rui Qu, Yanfeng Xie, Sheng Liu

**Affiliations:** 1 School of Mechanical Engineering, Hunan Institute of Engineering, Xiangtan 411104, China; qinbo@hnie.edu.cn (B.Q.); 17369283970@139.com (R.Q.); m18873404170@163.com (Y.X.); 2 Hunan Provincial Key Laboratory of Vehicle Power and Transmission System, Hunan Institute of Engineering, Xiangtan 411104, China

**Keywords:** dissimilar magnesium alloy, TIG welding, numerical simulation, temperature field

## Abstract

The welding experiments and numerical simulation analysis of dissimilar magnesium alloy AZ61-AM60 were carried out by TIG and A-TIG methods. The mathematical model of welding pool under three-dimensional transient moving heat source has been established, and the temperature field has been numerically simulated. The influence of welding process parameters on the surface forming quality of welded joints has been discussed. The simulation results show that temperature field distribution of dissimilar magnesium alloy AZ61-AM60 during the TIG welding process presents a certain asymmetry and the shape distribution of the melting field on both sides of the molten pool is asymmetrical. When A-TIG welding was coated with activating flux, the surface of the molten pool is ingot-shaped. These simulation results are verified through experiment investigation. The consistency between the experimental results and the simulation results reveals the variation law of temperature field and molten pool shape in the welding process, which provides an effective guidance for the optimization of welding process parameters of dissimilar magnesium alloys.

## 1. Introduction

With the expanding and deepening of the lightweight structural materials application field, magnesium alloys have attracted tremendous interest due to its low density and high specific strength [1,2]. However, the applications of complex structural components of magnesium alloy are limited as the welding of magnesium alloy joints is difficult [3]. With the increasing variety of new magnesium alloys, the welding of new magnesium alloy materials, especially dissimilar magnesium alloys, has attracted increasing attention. In order to obtain a high-quality magnesium alloy welded joint, reliable and effective welding technologies have been developed for magnesium alloys in recent years [4,5]. The developed welding methods include laser welding [6], friction stir welding [7], and laser–tungsten inert gas hybrid welding [8].

Tungsten inert gas shielded welding (TIG) has become one of the most effectively used welding methods for bonding magnesium alloys, which can achieve concentrated heating, small deformation of welding parts, high arc stability, excellent weld formation, and high mechanical strength of welded joints [9,10,11]. Shen and Xu [12] studied the effects of preheat treatments on the microstructure and mechanical behavior of AZ61 magnesium alloy joints by the TIG welding. The results indicated a positive correlation between the microhardness of the fusion zone (FZ) and the ultimate tensile strength of the welded joints with the preheat process. In addition, the TIG welding technology integrated with the long-wave ultraviolet A (UVA) treatment promoted the grain refinement of the magnesium alloy joints [13]. However, cracks, too wide heat affected zone, and coarse grains were prone to occur in the welding process. Zhang et al. [14] purposed a new route, named AC/DC mixed tungsten inert gas arc (MIX-TIG) welding, to improve the welding quality of magnesium alloy.

Although the TIG and A-TIG methods have become the common methods in recent magnesium alloy welding, few reports have been reported on their application in the welding of dissimilar magnesium alloys. Moreover, the optimizing and improving TIG welding process of dissimilar magnesium alloys by numerical simulation method is very rare. Numerical simulation is a powerful tool to analyze quantitatively the complicated multi-physical phenomena in welding dissimilar magnesium alloys [15,16,17,18]. For typical instance, Yuan et al. [19] investigated the temperature–stress distribution and generation law of liquation cracking in circular-patch welding of dissimilar Mg alloys. Numerical simulation results of temperature–stress field visually display stress distribution and peak value, and evaluate the susceptibility to liquation cracking of Mg alloys’ welding. Moreover, heat treatment before welding and controlling melting zone temperature can significantly reduce the adverse effect of liquefaction cracking. Although studies have focused on the simulation of temperature field and its related field-effect of dissimilar magnesium alloy welding, there are few studies on the dynamic temperature field and molten pool flow during magnesium alloy welding, which is undoubtedly crucial to the mechanical strength of dissimilar magnesium alloy welded joints. In addition, welding technology selection directly affects the generation of heat and the flow of materials and further determines the dynamic morphology and flow field state of welding pool, which are of great significance for the optimization of welding process [20].

In order to reveal the influencing factors of the welding joints quality and improve the structural reliability of dissimilar magnesium alloys, the welding experiments and numerical simulation analysis of dissimilar magnesium alloy AZ61-AM60 are carried out by TIG and A-TIG methods in this paper. A mathematical model of welding pool under three-dimensional transient moving heat source has been established, and the temperature field has been numerically simulated. The influence of welding process parameters on the surface forming quality of welded joints has been discussed.

## 2. Experiment

### 2.1. Welding Base Metal

**Figure 1:**
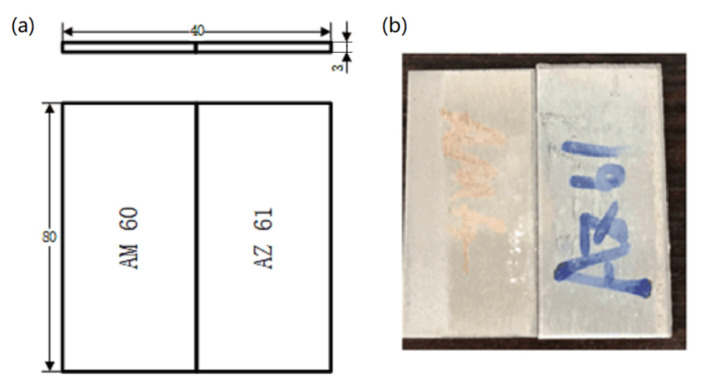
Welding sample schematic diagram. (**a**) Two dimensional diagram and (**b**) physical photo.

The welding materials used in this paper are dissimilar materials. The welding base metal is magnesium alloy AZ61-AM60, and the material size is 80 mm × 40 mm × 3 mm. The schematic diagram of the sample is shown in Figure 1, and the chemical composition of the base metal is shown in Table 1.

### 2.2. Coating Materials

**Figure 2:**
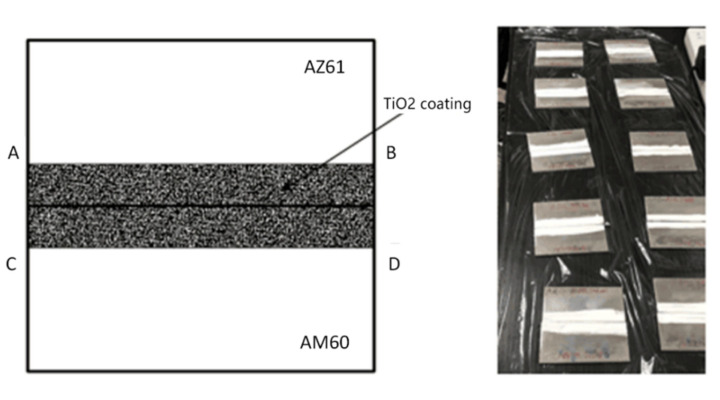
Schematic diagram of activating flux.

TiO_2_ was used as the active agent. An adequate amount of TiO_2_ was dissolved in anhydrous ethanol and stirred evenly, and the active agent was coated on both sides of the weld seam with a brush. The schematic diagram of coating is shown in Figure 2. In order to ensure uniform coating, as shown in AB and CD in Figure 2, a line 1 cm away from the end face of the sample was drawn on the surface along the welding seam. It is guaranteed that the active agent did not exceed this line during coating. Therefore, the coating area of a single sample was ensured to be 1 cm × 8 cm = 8 cm^2^. The surface coating density was calculated according to Equation (1), and the coating amounts were set as 5, 10, 15, and 20 mg/cm^2^, respectively:

(1) $$\rho =\frac{m_{1}-m_{0}}{S}$$

where *m*_1_ is the mass of the sample after coating, *m*_0_ is the mass of the sample before coating, and *S* is the coating area of the sample.

### 2.3. Experimental Methods and Equipment

**Figure 3:**
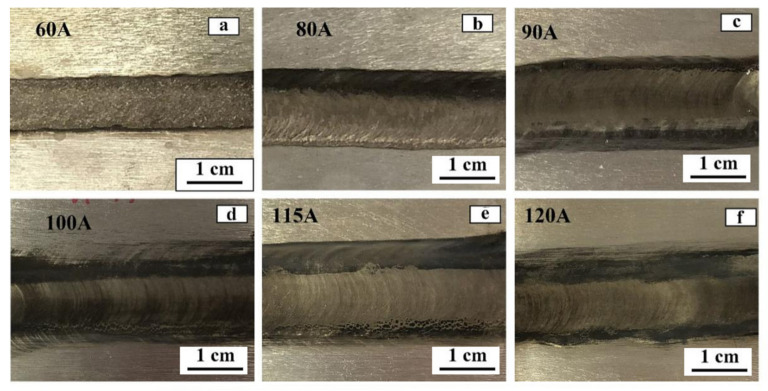
Macroscopic morphologies of TIG welding seams of AZ61-AM60 dissimilar magnesium alloy under different welding currents: (**a**) 60 A, (**b**) 80 A, (**c**) 90 A, (**d**) 100 A, (**e**) 115 A, and (**f**) 120 A.

The experiment used the AC/DC argon arc welding machine (AVP-360, OTC Industrial Co.,Ltd., Shaoxing, China) and adopted single-sided welding and double-sided forming as the welding process. In order to reduce the deformation of the welding, a fixture was used to fix the two horizontal plates before welding, as shown in Figure 3. Argon gas protection was used during the welding process. The dissimilar magnesium alloys were welded with an argon gas flow of 7.5 L/min. The welding voltage was set to a constant value of 20 V. The welding line energy can be adjusted by changing the welding current or welding speed according to Equation (2):

(2) $$L=\eta \frac{UI}{v}$$

where *L*, *U*, *I,* and *v* represent the welding line energy, welding voltage, welding current and welding speed, respectively; *η* is welding efficiency and set to 0.80 in this experiment.

### 2.4. Effects of Welding Current on TIG Welding Formation

Figure 3 shows the macroscopic morphology of TIG welding seam of alloy AZ61-AM60 under different welding current. It can be seen that, with the increase of the current, the front surface of the welding seam becomes smoother, and the welding path becomes more uniform and full. When the welding current is 120 A, there are no apparent defects such as pores, cracks and welding nodules in the welded joint.

**Figure 4:**
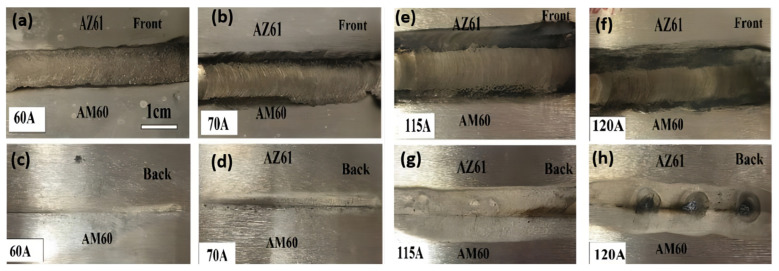
Macroscopic morphologies of front and back surface of AZ61-AM60 dissimilar magnesium alloy during TIG welding under different welding currents.

Figure 4 displays the macroscopic images of the face and root of AZ61-AM60 dissimilar magnesium alloy during TIG welding under a different welding current. As shown in Figure 4a, when the welding current is 60 A, the front side of the joint welding seam is well-formed. However, due to the low line energy (320 J/mm), the heat input of the molten pool is insufficient, and the base metal is not easy to melt, resulting in incomplete fusion and penetration of the backside and poor forming, as shown in Figure 4c. As welding current increases, the welding heat input and the melting width gradually increases. Under the welding current of 115 A, the joint welding seam is dense and the welding width is uniform, without bites, pores, and cracks. As shown in Figure 4e,g, the welding width increases to 11 mm, both the face and root of the welding seams are well-formed. When the current continues to increase to 120 A, the line energy reaches 640 J/mm. Due to excessive welding heat input, the joint welding seam is directly penetrated, and the welding cannot be formed, as shown in Figure 4h.

Based on the tested parameters and comparing the macroscopic morphology of the welding seam, it is found that the welding current will evidently affect the welding formation during TIG welding of dissimilar magnesium alloys AZ61 and AM60. Therefore, selecting an appropriate welding current can obtain a good welding formation. When the welding current is set to 115 A, the appearance quality of the welding formation is the best. The above results can provide an experimental basis for setting the value of welding current in the subsequent numerical simulation.

### 2.5. Effects of Welding Current on the Formability of A-TIG Welding Joints

**Figure 5:**
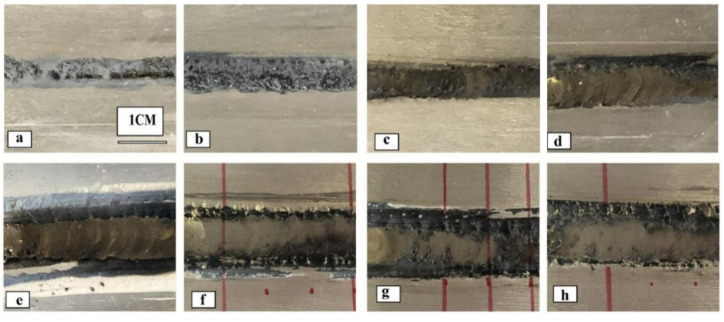
Macroscopic morphologies of AZ61-AM60 dissimilar magnesium alloy during A-TIG welding under different welding currents: (**a**) 60 A, (**b**) 70 A, (**c**) 80 A, (**d**) 90 A, (**e**) 100 A, (**f**) 110 A, (**g**) 115 A, and (**h**) 120 A.

Figure 5 shows the macroscopic morphology of AZ61-AM60 alloy during A-TIG welding at different welding currents. After welding, a small amount of activating flux TiO_2_ remains on different parts of the welding seam. There are bits of black spot slag on the welding surface, which is the most obvious at the welding current of 115 A. There are no obvious surface defects such as pores and cracks. As the welding current increases, the heat input and the width of the welding seam increase.

### 2.6. Effects of Welding Current on the Microstructure of Welding Seam

**Figure 6:**
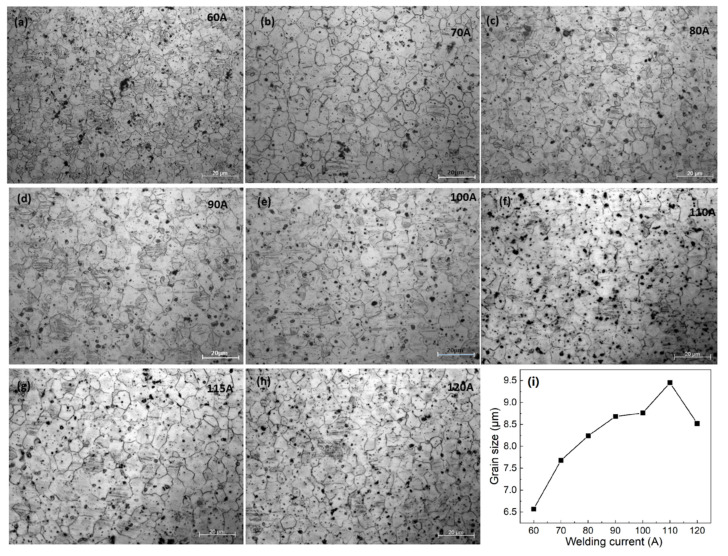
Microstructures of TIG welding seam under different welding currents. (**a**) 60 A, (**b**) 70 A, (**c**) 80 A, (**d**) 90 A, (**e**) 100 A, (**f**) 110 A, (**g**) 115 A, (**h**) 120 A, (**i**) grain size of TIG welding versus welding current.

**Figure 7:**
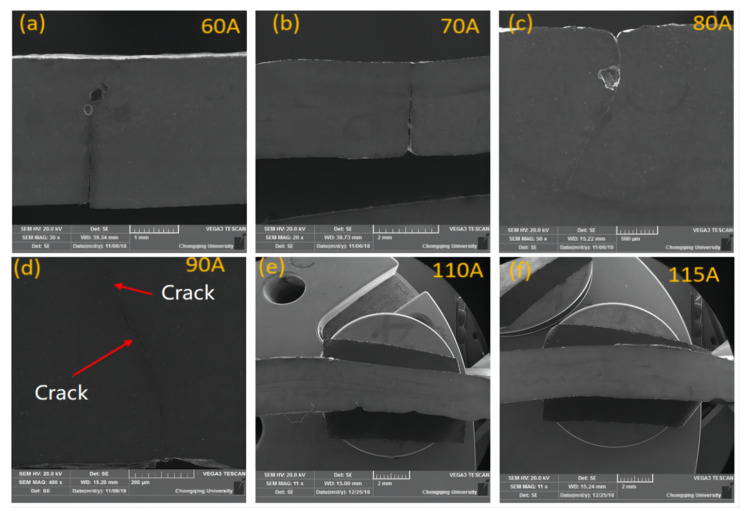
Cross section of TIG welding seams under different welding currents. (**a**) 60 A, (**b**) 70 A, (**c**) 80 A, (**d**) 90 A, (**e**) 110 A, (**f**) 115 A.

The microstructure of AZ61-AM60 alloy TIG weld under the different welding current is shown in Figure 6a–h. The microstructure of the welding seam is equiaxed crystal, and there are obvious twin crystals and point-like precipitates in the grains. With the increase of welding current, the average grain size of the welding seam first increases and then decreases, as shown in Figure 6i. Under the welding current of 60 A, the average grain size of 6.57 μm is the smallest. Due to the weak welding line energy under 60 A–90 A current range and the insufficient heat input of the molten pool, the weld cannot be fully penetrated (as shown in Figure 7). When the welding current reaches 100 A, the heat input is sufficient to penetrate 3 mm thick workpieces. With the increase of current, the grain size reaches the maximum value at 110 A. After that, as the current increases, the existence time of molten pool becomes longer, and the cooling rate after welding becomes slower.

**Figure 8:**
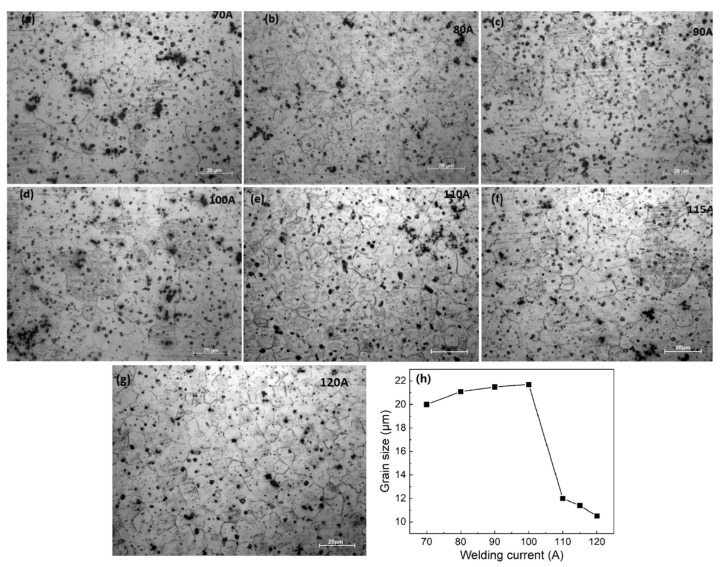
Microstructures of A-TIG welding seam under different welding current conditions (**a**) 70 A, (**b**) 80 A, (**c**) 90 A, (**d**) 100 A, (**e**) 110 A, (**f**) 115 A, (**g**) 120 A, (**h**) grain size of A-TIG welding seam versus welding current.

Figure 8a–g shows the microstructure of the AZ61-AM60 alloy A-TIG welding seam under different welding currents. It can be seen that there are uniform equiaxed crystals in the weld zone. With the increase of current, the grain size increases first and then decreases, as shown in Figure 8h. When the current is 100 A, the grain size reaches the maximum with an average size of 21.7 μm. The distribution of precipitates is relatively uniform. Compared with TIG welding with the same welding process, the grain size of weld zone increases, indicating that the addition of active agent has a great influence on the grain size of weld zone. In addition, the heat input and cooling rate during the welding process are the main factors affecting the microstructure of the welding seam.

### 2.7. Analysis of Welded Joint Mechanical Property

**Figure 9:**
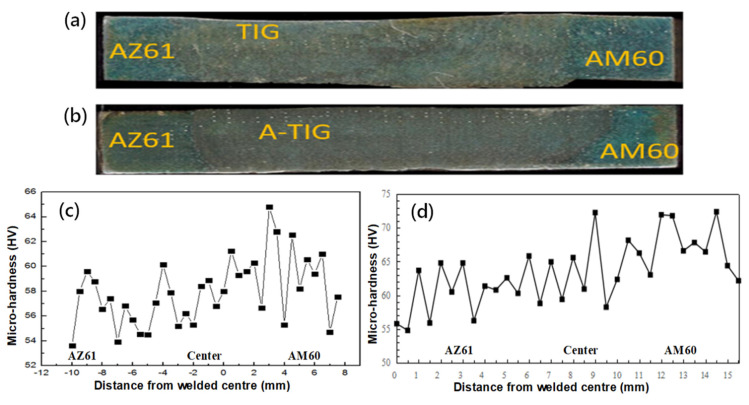
Diagrams of microhardness measurement of TIG (**a**) and A-TIG (**b**) welded joints; (**c**) the microhardness distribution of (**c**) TIG and (**d**) A-TIG welded joint under welding, respectively.

Figure 9 shows the microhardness distribution of TIG welded joints under welding current of 115 A and welding speed of 180 mm/min. The schematic diagram of microhardness measurement of TIG welded joints is shown in Figure 9a. As can be seen from Figure 9c, AZ61 base metal has a lower average hardness than it does on the AM60 side. In the TIG welded joint, the hardness of HAZ (heat affected zone) is lower than that of the base metal and the weld zone, which is caused by the coarse grains in the HAZ. In the center of the welding seam, the hardness of the side close to AM60 alloy is higher than that of the other parts. From the center of the welding seam to the HAZ, the hardness value first decreases and then increases, and then decreases. The change of microstructure can be judged from the change of hardness: coarse equiaxed crystals appears in the center of the welding seam, followed by columnar crystal, and the hardness decreases; further outward, fine equiaxed crystal in the area near the fusion line, and the hardness increases again. Outside the fusion line, the base metal grains in HAZ are coarse, and the hardness decreases again. This result is different from the dissimilar joints of P91 and P22 steels [21]. Since AM60 alloy contains no Zn and there is no MgZn compound, more Mg_17_Al_12_ compounds are precipitated during the solidification of the molten pool, resulting in higher hardness on the AM60 side. While Mg_17_Al_12_ and MgZn compounds coexist in AZ61, and the composition of Mg_17_Al_12_ is relatively small, its contribution to hardness is therefore lower.

**Figure 10:**
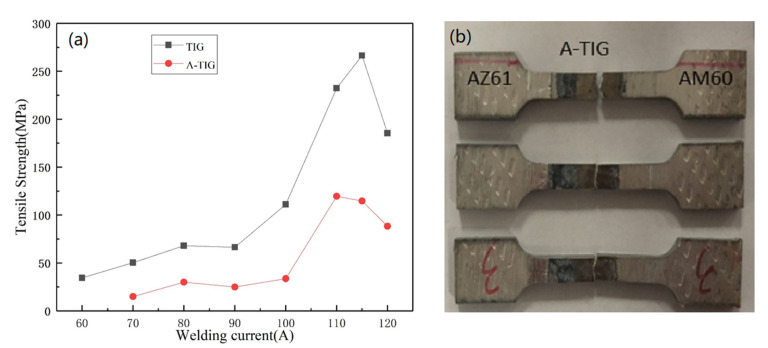
(**a**) Tensile strength of TIG and A-TIG welding joints under different welding currents; (**b**) fracture location of A-TIG welded joints of the AZ61-AM60 dissimilar magnesium alloy.

The tensile strength curves of TIG and A-TIG welded joints under different welding currents are given in Figure 10a. It can be seen that, with the increase of current, the strength of both TIG and A-TIG welded joints increases first and then decreases, and the variation trends are consistent. At 115 A, the strength of TIG welded joints reaches the maximum value of 266.6 MPa, while the strength of A-TIG welded joint achieves the maximum value of 119.7 MPa at 110 A. Figure 10b shows the fracture location of A-TIG welded joints of the AZ61-AM60 dissimilar magnesium alloy. It can be seen that, for all the tensile tests, the joints fractured on the AM60 side. Since the hardness fluctuates greatly on the AM60 side, the deformation has difficulty occurring in the area with high hardness, and the plastic deformations are concentrated in the weak area with low hardness.

## 3. Numerical Simulation

### 3.1. Hypothesis of the Welding Model

There are many factors influencing TIG welding process, such as heat transfer, radiation, convection, and phase transformation. Before simulation, the following assumption about the TIG welding process is made:

(1) Assuming that the liquid metal in the molten pool is an incompressible Newtonian fluid that behaves as the laminar flow;

(2) Assuming that the upper surface of the molten pool is flat, which can simplify the calculation process and ensure that the calculation results are not distorted;

(3) For the forces driving the liquid metal flow in the molten pool, only buoyancy, surface tension, and electromagnetic force are considered, while arc pressure and positive pressure caused by surface curvature is ignored;

(4) The distribution of current density and heat flux density of the welding arc is assumed to be double ellipsoid heat source;

(5) Assuming that the thermophysical properties of the material only vary with temperature.

### 3.2. Geometric Model and Meshing

**Figure 11:**
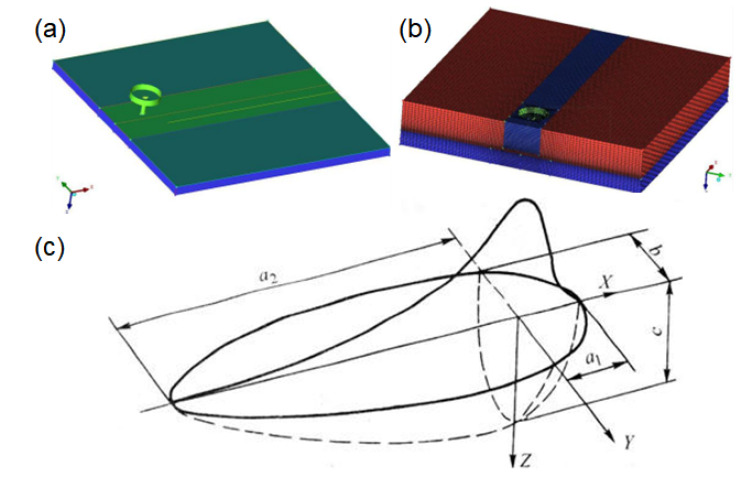
(**a**) Geometric model of numerical simulation of welding, (**b**) meshing of the model and (**c**) double ellipsoid model.

The numerical simulation of TIG welding pool of dissimilar magnesium alloy is carried out. Numerical simulation of dissimilar metals is special. It is essential to build a complete geometric model, as shown in Figure 11a. In this case, the grids of this simulation body are all structured grids (cells), and the number is about 580,000. Among them, the number of face grids is about 180,000. The total number of nodes is about 630,000, and the minimum grid mass is 0.64, as shown in Figure 11b.

### 3.3. Establishment of Welding Heat Source

The accuracy of the temperature field calculation depends on the heat source model, so the establishment of the TIG welding heat source model suitable for dissimilar materials is a prerequisite for accurate simulation of the welding process.

For TIG welding, the establishment of the heat source model has experienced from the initial fixed arc to moving arc, from steady state to quasi-steady state and then to transient state, from 2D to 3D, from the surface heat source (Gaussian heat source) to the double elliptic heat source and then to double ellipsoid heat source. The final model is getting closer and closer to reality. The double ellipsoid model used in this paper is shown in Figure 11c.

Assuming that the semi-axis lengths of the front ellipsoid in the *X*, *Y*, and Z directions are specifically *a*_1_, *b*, and *c*, the corresponding heat source distribution is as follows:

(3) $$q_{1}\left(x,y,z\right)=\frac{6\sqrt{3}\left(f_{1}Q\right)}{a_{1}bc\pi \sqrt{\pi }}\exp\left(-\frac{3x^{2}}{a_{1}{}^{2}}-\frac{3y^{2}}{b^{2}}-\frac{3z^{2}}{c^{2}}\right),\;x\geq 0$$

Assuming that the semi-axis lengths of the rear ellipsoid in the *X*, *Y*, and Z directions are specifically *a*_2_, *b*, and *c*, the corresponding heat source distribution is as follows:

(4) $$q_{2}\left(x,y,z\right)=\frac{6\sqrt{3}\left(f_{2}Q\right)}{a_{2}bc\pi \sqrt{\pi }}\exp\left(-\frac{3x^{2}}{a_{2}{}^{2}}-\frac{3y^{2}}{b^{2}}-\frac{3z^{2}}{c^{2}}\right),\;x\geq 0$$

Among them, *η* is the effective power coefficient of the arc, taking 0.8; *I* is the welding current; the welding voltage *U* is 20 V; and σ_*q*_ is the effective heat flow density distribution radius.

### 3.4. Processing of Boundary Conditions

The initial temperature of the workpiece is set to 298.15 K (ambient temperature), and the boundary conditions of the upper surface of the workpiece are as in:

(5) $$k\left(\frac{\partial T}{\partial z}\right)=q_{arc}-a_{c}(T-T_{a})-\varepsilon \sigma_{b}(T^{4}-T_{a}^{4})$$

(6) $$\frac{\partial u}{\partial z}=\frac{\partial v}{\partial z}=w=0$$

Among them, $a_{c}$ is the heat transfer coefficient between the material and the air, $T_{a}$ is the air temperature, *ε* is the gray level of the material, $\sigma_{b}$ is the Boltzmann constant, and *u*, *v* and *w* denote the directions of the *x*, *y,* and *z*-axes, respectively.

The boundary conditions of the model end face are:

(7) $$k\left(\frac{\partial T}{\partial z}\right)=-a_{c}(T-T_{a})-\varepsilon \sigma_{b}(T^{4}-T_{a}^{4})$$

(8) u=v=w=0

### 3.5. Addition of Driving Forces

The driving forces in the welding process simulation are performed by the source term, and the specific driving force types are as follows:

(1) Buoyancy

(9) $$S_{b}=\rho g\beta (T-T_{m})$$

where β is the coefficient of thermal expansion, $T_{m}$ is the melting point of AZ61, and the buoyancy acts only in the *z*-axis direction.

(2) Electromagnetic force

(10) $$\vec{F_{y}}={\left(\vec{J}\times \vec{B}\right)}_{y}=-\frac{\mu_{0}I^{2}}{4\pi^{2}\sigma_{j}^{2}r}\exp\left(-\frac{3r^{2}}{\sigma_{j}^{2}}\right)\left[1-\exp\left(-\frac{3r^{2}}{\sigma_{j}^{2}}\right)\right]{\left(1-\frac{z}{L_{z}}\right)}^{2}\frac{y}{r}$$

(11) $$\vec{F_{x}}={\left(\vec{J}\times \vec{B}\right)}_{x}=-\frac{\mu_{0}I^{2}}{4\pi^{2}\sigma_{j}^{2}r}\exp\left(-\frac{3r^{2}}{\sigma_{j}^{2}}\right)\left[1-\exp\left(-\frac{3r^{2}}{\sigma_{j}^{2}}\right)\right]{\left(1-\frac{z}{L_{z}}\right)}^{2}\frac{x}{r}$$

(12) $$\vec{F_{z}}={\left(\vec{J}\times \vec{B}\right)}_{z}=-\frac{\mu_{0}I^{2}}{4\pi^{2}\sigma_{j}^{2}r}\left[1-\exp\left(-\frac{3r^{2}}{\sigma_{j}^{2}}\right)\right]{\left(1-\frac{z}{L_{z}}\right)}^{2}$$

where $\vec{J}$ is the current density vector, $\vec{B}$ is the magnetic flux vector, $\sigma_{j}$ is the distribution parameter of the welding current, and $L_{z}$ is the thickness of the workpiece.

(3) Surface tension

As the main driving force of welding pool, surface tension largely determines the flow pattern of molten metal. The expression of surface tension is as follows:

(13) $$-\mu \frac{\partial u}{\partial z}=\left(\frac{\partial \gamma }{\partial T}\right)\left(\frac{\partial T}{\partial x}\right)$$

(14) $$-\mu \frac{\partial u}{\partial z}=\left(\frac{\partial \gamma }{\partial T}\right)\left(\frac{\partial T}{\partial y}\right)$$

Among them, *μ* is viscosity, *γ* is surface tension, and *∂γ/∂T* is the temperature coefficient of surface tension. Many researchers have found that the temperature gradient of surface tension is very important. However, the actual surface tension coefficient of various materials is very difficult to obtain in the commonly studied temperature range. Therefore, the surface tension coefficient is taken as a constant in this study.

### 3.6. Determination of Material Thermal Physical Parameters

The low-temperature physical property parameters of the materials in this paper are referred to the relevant literature, and the high-temperature physical property parameters are obtained by extrapolation. Among them, the density of AZ61 is 1.8 g/cm^3^, the liquidus temperature is 883 K (610 °C), the solidus is 798 K (525 °C); the liquidus of AM60 is 888 K (615 °C), and the solidus is 818 K (545 °C). Some parameters are shown in Table 2 and Table 3.

## 4. Results and Discussion

### 4.1. Simulation of Temperature Field

**Figure 12:**
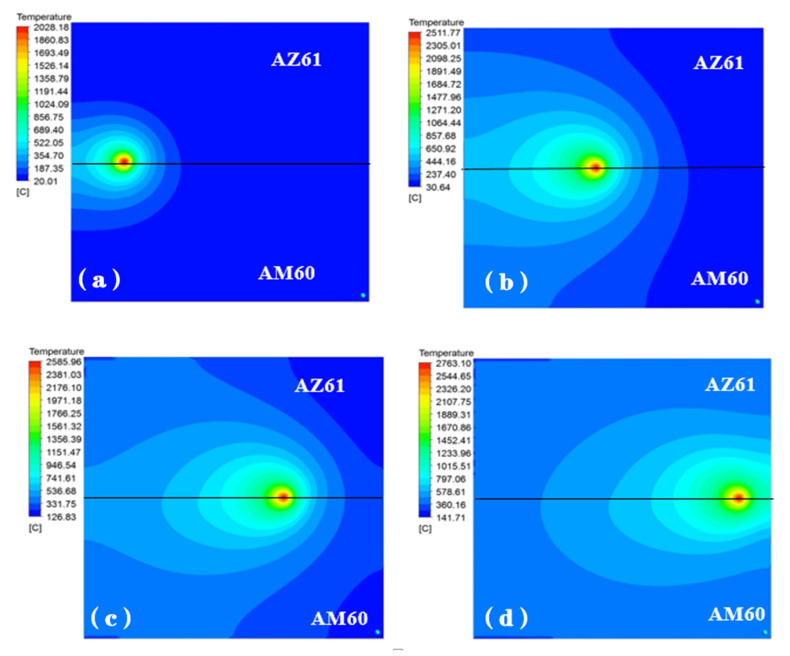
Temperature field distribution on the upper surface of the molten pool at the different time with the welding current of 115 A: (**a**) 1.5 s, (**b**) 8.5 s, (**c**) 14.5 s, (**d**) 20.5 s.

Figure 12 shows the temperature field distribution of dissimilar magnesium alloy AZ61-AM60 during TIG welding when the welding current is 115 A. As the heat source moves, the molten pool continues to move forward. When the welding time is 1.5 s, the highest temperature of the workpiece reaches 2028 °C. At this time, there is no obvious difference in temperature distribution on two sides of AZ61 and AM60, and the shape of molten pool is basically symmetrical. As time goes on, when the welding time is 8.5 s, the range affected by the heat source gradually expands. The area affected by the heat source on AZ61 side is wider than that on the ZK60 side, and the highest temperature reaches 2512 °C. When the welding time is 14.5 s, the arc becomes stable and the highest temperature reaches about 2585 °C. When the welding time is 20.5 s, the workpiece temperature reaches 2763 °C.

It can be seen from the temperature cloud diagram in Figure 12 that, during the welding process, the temperature at the center of the heat source is the highest. As the heat source moves forward, the effective heating area of the welded workpiece increases gradually due to the influence of the acceptor heat source being heated. When heat source moves, the distribution of the temperature field will also change. As the arc reaches stability, the maximum temperature and shape of the molten pool are basically fixed. It can be seen from Figure 12d that the temperature fields on both sides of AZ61 and AM60 are asymmetrically distributed. Since the thermal conductivity of AM60 is larger than that of AZ61, the cooling rate of AM60 is faster and the area is larger.

**Figure 13:**
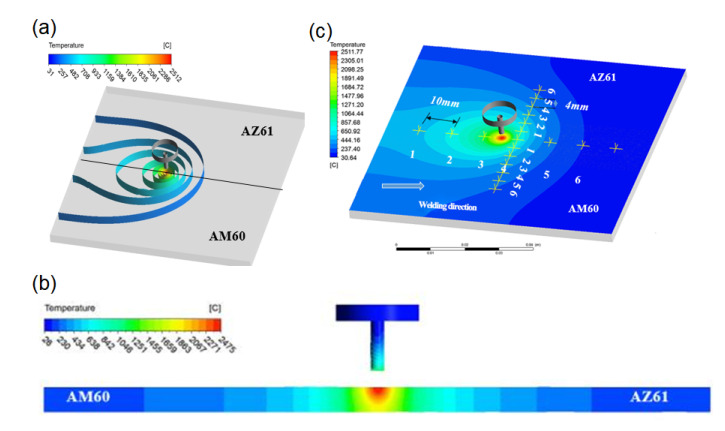
(**a**) Distribution of isothermal surface at 8.5 s, (**b**) cross-section view of the welding parts at 8.5 s and (**c**) schematic diagram of selected thermal cycle nodes.

The temperature field can be further understood by taking the isotherm distribution of different regions at the same time, as shown in Figure 13a. It can be seen that, in the area directly affected by the heat source, due to the good thermal conductivity of magnesium alloy and the extremely fast heating speed, the isotherm distribution is dense close to the heat source, and sparse in the place far from the heat source.

The cross-section of the welding parts at 8.5 s is studied, and the cross-section of the molten pool is shown in Figure 13b. It can be seen from the analysis that the welding part is completely penetrated. Compared to the melting width of the lower surface, the melting width of the upper surface of the welding part is relatively wider, which is very similar to the actual welding shape. Since magnesium alloys on two sides have different physical properties, and the molten pools on two sides have basically symmetrical distribution. AM60 has a larger thermal conductivity and dissipates heat faster than AZ61, so the width of the low temperature (blue) region of AM60 is smaller than the low temperature (blue) region of AZ61. This means that the temperature gradient of the AM60 region is larger, which is consistent with the actual situation.

**Figure 14:**
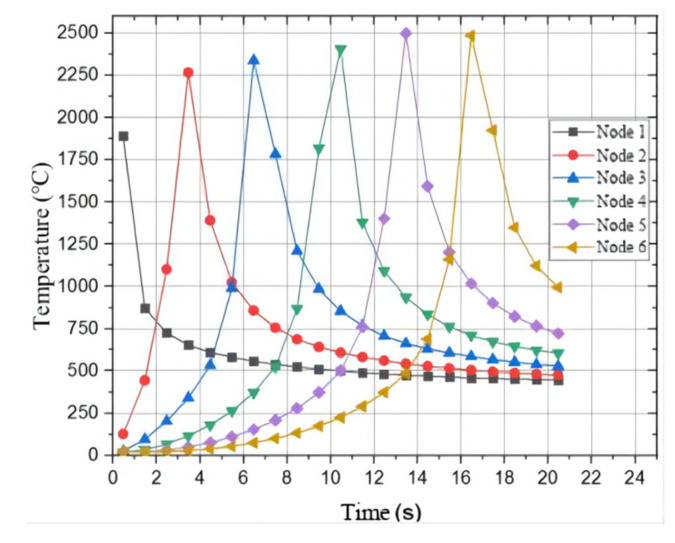
The thermal cycle curves of selected nodes at the center of welding seam along the welding direction.

In order to obtain the distribution of temperature fields on both sides of AZ61 and AM60 dissimilar magnesium alloys during TIG welding, typical nodes were selected for thermal cycle curve analysis. First, the nodes were selected at equal intervals along the welding direction and then the thermal cycle curve was calculated. The distances between the selected welding nodes and the welding starting point are 10 mm, 20 mm, 30 mm, 40 mm, 50 mm and 60 mm, respectively. The corresponding thermal cycle curves are drawn and the nodes are defined as 1–6 respectively. The temperature curves of each node are shown in Figure 13c, and their thermal cycling curves are shown in Figure 14.

It can be seen from Figure 14 that the welding heat source keeps moving, and the nodes reach the highest temperature in turn. When the heat source is close to a node, the temperature rises first and then falls, and the change speed is very fast. This shows that the heat transfer rate of TIG welding is very fast in magnesium alloy welding, and the magnesium alloy can be quickly melted, which is also one of the reasons to ensure that the welding surface is smooth and flat. As time progresses, the following nodes reach the highest temperature in turn, indicating that the closer to the heat source, the highest temperature is reached first. At the same time, the temperature increases slightly as time goes on, and tends to be stable after node 5 and remains about 2500 °C. This is consistent with the actual test, the arc is unstable at the beginning, and gradually tends to be stable with the movement of heat source. By observing the surface quality of the welding seam, it can be found that the surface forming quality of welding part is better at about 5–10 mm away from the end face of the beginning arc, which is also related to the thermal cycle characteristics of each point on the welding part.

**Figure 15:**
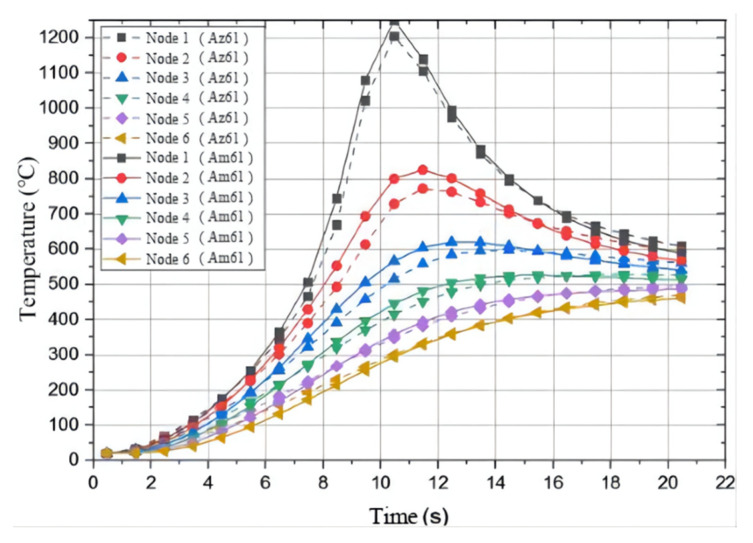
Thermal cycle curves of nodes taken on the AZ61 side and AM60 side perpendicular to the welding seam.

In order to further explore the characteristics of the welding temperature field of dissimilar magnesium alloys, six nodes were selected in the vertical direction on the AZ61 side and the AM60 side, and then the thermal cycle curve analysis was carried out. The nodes were selected at 4 mm, 8 mm, 12 mm, 16 mm, 20 mm, and 24 mm away from the center of the welding seam on both sides, denoted as 1–6, respectively. Their thermal cycle curves are shown in Figure 15. It can be seen that there are some differences in the maximum temperature of each node on both sides of the same distance from the center of the welding seam. The solid line data are the temperature value of AM60, and the dotted line data are the temperature value of AZ61. Furthermore, in the heating stage, the temperature of the AM60 side is higher than that of the AZ61 side. Because of the difference in thermal physical properties of both sides, AM60 has a higher thermal conductivity, its temperature rises faster than AZ61 during heating, and the temperature gradient is larger. In the cooling stage, the cooling speed of AM60 is also faster than that of AZ61. An equilibrium point was reached around 16 s, after which the temperature of AZ61 was higher than that of AM60, and both showed a decreasing trend. It can be seen that, during TIG welding of AZ61 and AM60, the temperature of the molten pool is asymmetrically distributed, and the amount of melting metal on both sides is also different. This is consistent with the actual situation.

### 4.2. Simulation of Morphology and Flow Field of Molten Pool

(1) Morphological characteristics of molten pool

**Figure 16:**
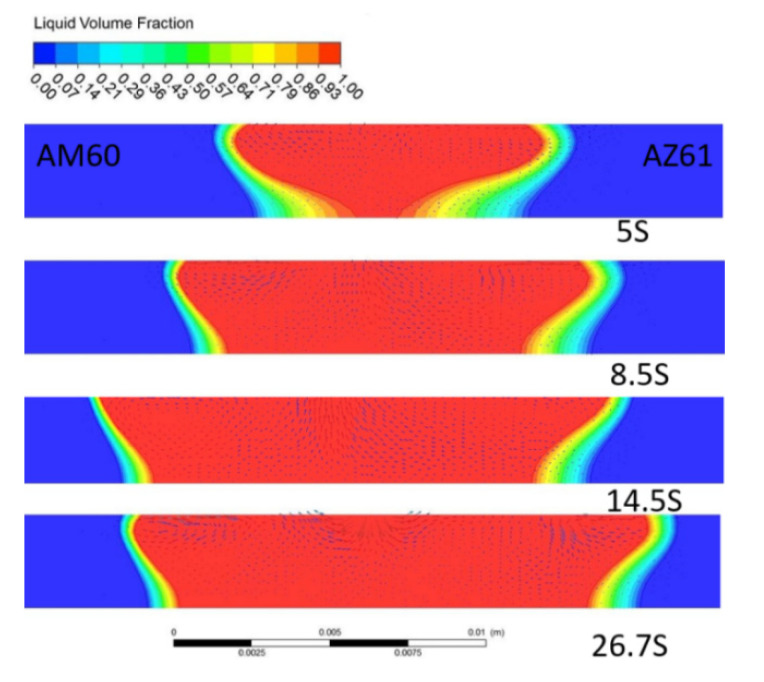
Welding pool shape at different times.

Figure 16 displays the morphological characteristics of the molten pool at different times. With the passage of time, the width of the molten pool gradually increases. When the time is 5 s, the heat source moves to the 15 mm position, the melting width of the upper surface of the molten pool is 9.05 mm; at a time of 8 s, the width of the upper surface is 12.71 mm, and at the time of 14.5 s, the width reaches 16.51 mm; then, it remains stable. At the end of welding, the melting width on the upper surface of the pool is 16.50 mm, which is almost the same as the melting width at 14.5 s, but there is a slight difference in shape. The overall shape of the molten pool cross-section shows that the upper surface of the pool is large, that is, the melting width is large, and the melting width on the lower surface is small. It exhibits an asymmetric “bowl shape”. The temperature range of the AZ61 side is wider, and that of the AM60 side is narrower; this means that the temperature gradient of AZ61 is smaller and the cooling process is slower, resulting from the small thermal conductivity of AZ61.

(2) Analysis of molten pool flow field

**Figure 17:**
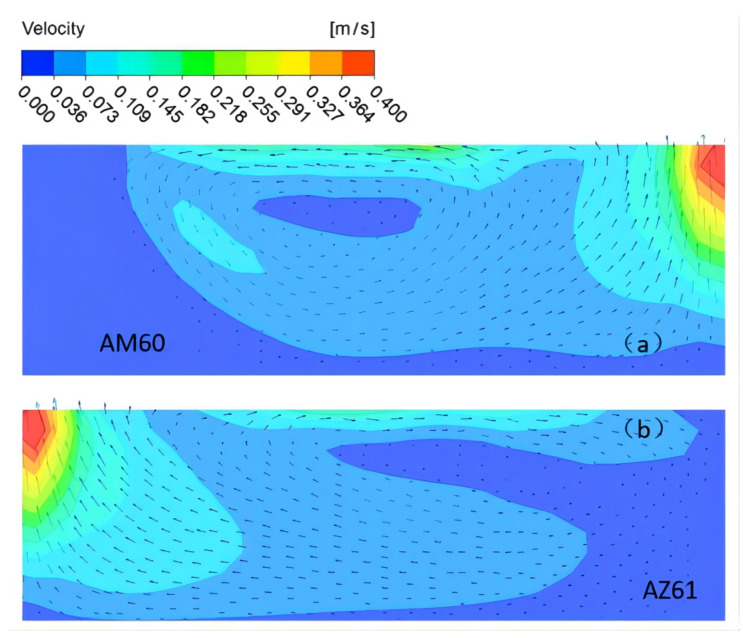
Velocity field of the molten pool perpendicular to the welding direction under the combined action force.

**Figure 18:**
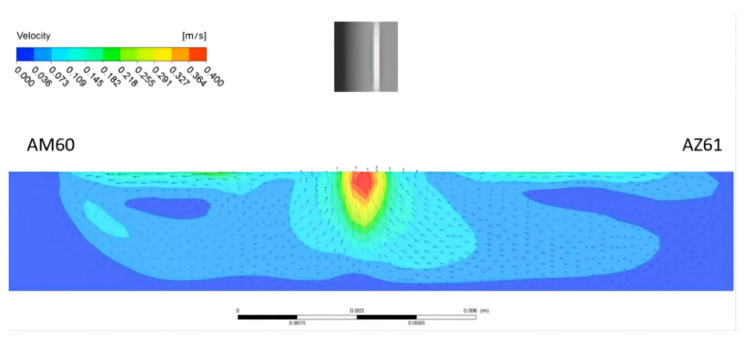
Enlarged view of velocity field of the molten pool perpendicular to the welding direction.

**Figure 19:**
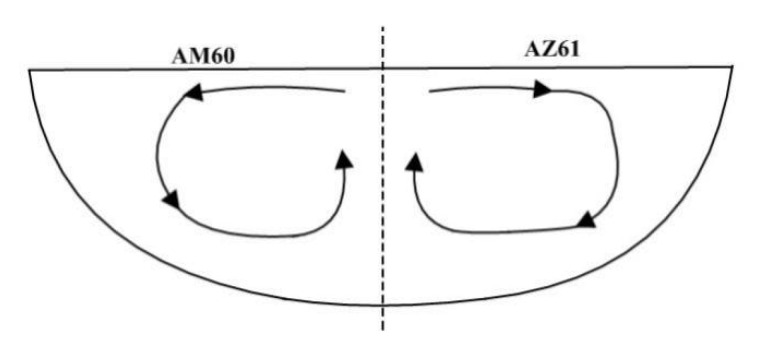
Model of the welding molten pool perpendicular to the welding direction.

With the welding current of 115 A, welding speed of 180 mm/min, and welding time of 7 s, under the combined action of buoyancy, electromagnetic force, and surface tension, the simulation of the velocity field of the molten pool perpendicular to the welding direction is shown in Figure 17. It can be seen that the position of the maximum flow velocity of the molten pool is directly below the heat source, and the maximum flow velocity is 0.4 m/s. Its enlarged view is shown in Figure 18. On the AM60 side, as shown in Figure 17a, first the upper surface of metal in the molten pool gradually moves from the center of the molten pool to the surrounding, then flows downward along the boundary of the molten pool, finally flowing toward the center of the molten pool when it is close to the center line of the welding seam. The movement direction of the AZ61 side is just symmetrical with that of the AM60 side, but the shape of the molten pool is asymmetrical. The model diagram of the flow of the welding molten pool is shown in Figure 19.

**Figure 20:**
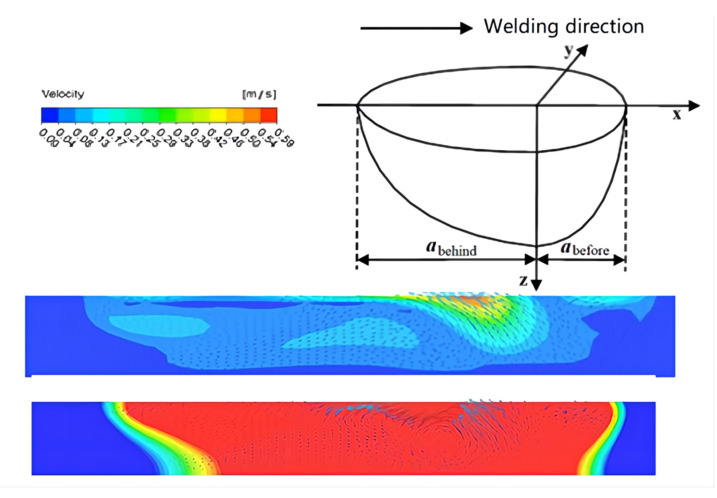
Morphology of molten pool along the welding direction under the combined action force and the schematic diagram of the double ellipsoid model of the heat source.

Figure 20 shows the morphology of the metal molten pool along the welding direction. The temperature gradient of the molten pool at the front of the welding direction is larger, and the heat conduction is faster. At the back of the molten pool, the temperature gradient of the molten metal is smaller and so the liquid flow is slower. The temperature distribution law of the heat source is in good agreement with the double ellipsoid model.

### 4.3. Simulation Results and Experimental Verification

**Figure 21:**
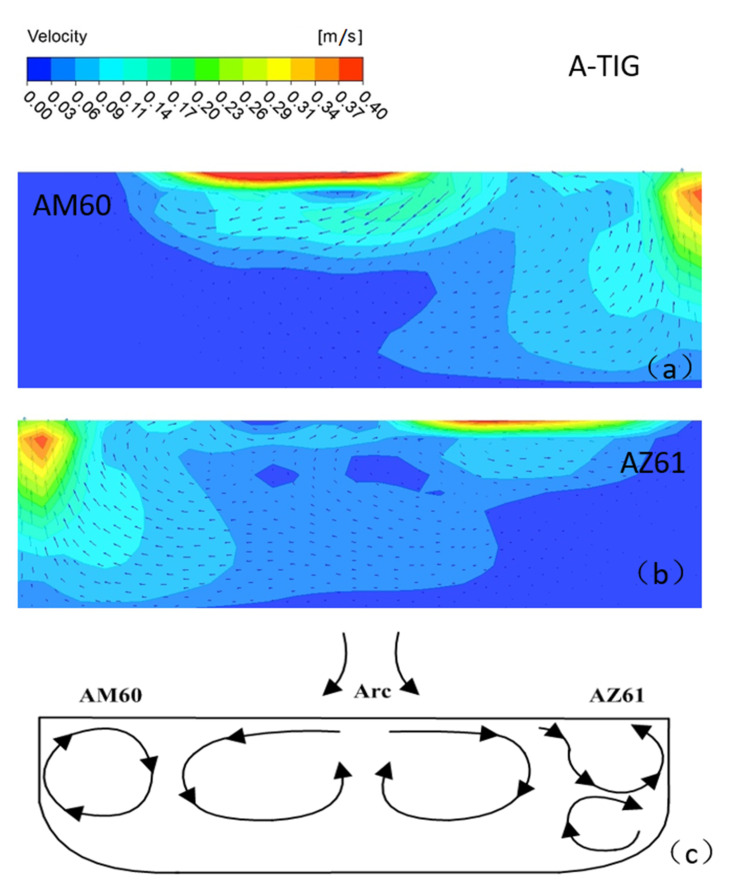
Distribution of velocity field of molten pool during A-TIG welding: (**a**) AM60, (**b**) AZ61, (**c**) model.

**Figure 22:**
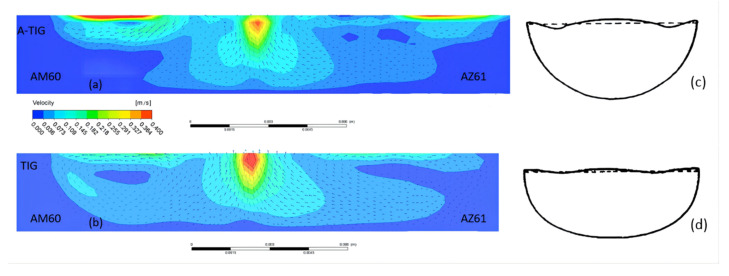
Distribution and model of velocity field of molten pool cross-section during TIG and TIG welding at 115 A: (**a**) AM60 during A-TIG welding, (**b**) AM60 during TIG welding, (**c**) welding pool model of AM60 A-TIG, (**d**) welding pool model of AM60 TIG.

Figure 21 shows the velocity field distribution of the cross-section of the molten pool perpendicular to the welding direction during A-TIG welding of dissimilar magnesium alloys AZ61 and AM60. According to the studies of Xie et al. [24] and Wu et al. [25], during A-TIG welding with activating flux, the increase of penetration depth is mainly due to the influence of surface tension. Furthermore, the positive and negative temperature gradient has a great impact on the depth of the molten pool. Figure 22a shows the flow state of the metal molten pool on the AM60 side. The upper surface of the molten metal will gradually flow from the pool center to the surroundings. The cross-sectional flow field model is shown in Figure 22c. One part of the metal surface forms a small eddy current far from the center of the welding seam, and the other part near the center begins to flow down along the pool boundary. When approaching the AM60-AZ61 interface, the axis line of the welding heat source of liquid metal TIG begins to flow toward the center of the molten pool. On the AZ61 side, the flow direction of the molten pool area near the axis line is the same as that of the AM60 side. The liquid metal flows slowly from the center line of the molten pool to the surrounding area, producing two small eddy currents in the area far from the center.

The conditions of dissimilar magnesium alloys AZ61 and AM60 were compared under TIG and A-TIG welding. From Figure 22, it can be seen that, when A-TIG welding coated with activating flux is carried out (∂γ/∂T > 0), the surface of molten pool is ingot-shaped. Meanwhile, the penetration depth of the welding pool is large, and the welding seam is narrow, and two weld grooves similar to the bite edge appear at the edge of the welding seam. During TIG welding (∂γ/∂T < 0), the welding surface is flat, the molten pool is shallow, and the welding seam is wider, which is consistent with the conclusion of Paul et al. [26], as shown in Figure 22c,d.

**Figure 23:**
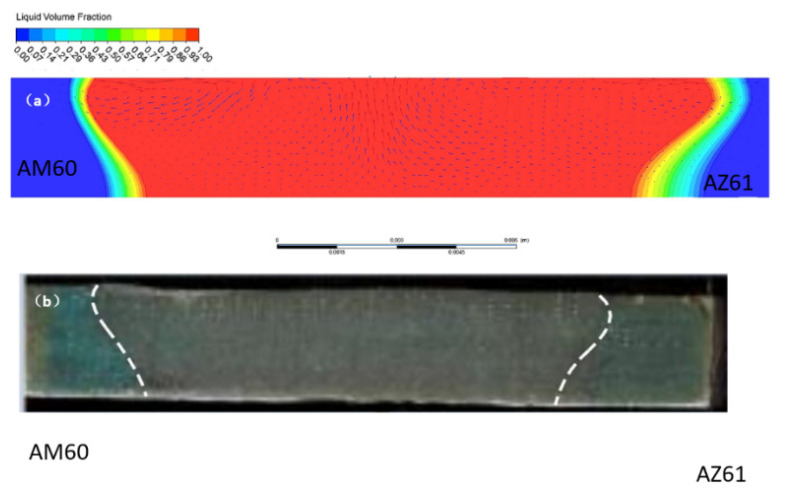
Comparison diagram of morphology simulation and experimental values of the TIG welding pool at current of 115 A: (**a**) simulation morphology, (**b**) experimental photo.

The mathematical model reliability of the welding process is usually verified by experiments, and its parameter settings are constantly revised to make it closer to the actual welding process. Figure 23 presents a comparison diagram of the TIG welding pool morphology simulation and experimental values when the welding current is 115 A. First of all, from the cross-section shape, the appearance of the two is generally consistent; the upper end face is wide, the lower end face is narrow, and the middle is transitioned in a smooth arc. They have similar contour and the cross-sections of the welding seam are well matched too. However, from the measurement results, the size of the upper end face achieved by the experiment is 10 nm, while the simulated size is 14.2 mm. The simulated value is larger than the actual value, which is caused by two factors: the selected large simulation efficiency value of 0.8 and the distortion of physical performance parameters for the high-temperature metal.

## 5. Conclusions

TIG (A-TIG) welding experiments were carried out on dissimilar magnesium alloys AZ61 and AM60, and the influence of welding current on the welding seam formation was studied. The mathematical model of welding pool has been established, and the welding process was numerically simulated. The following conclusions are drawn:

(1) By analyzing the TIG welding of dissimilar AZ61-AM60 alloys, the optimal process parameters were obtained. In TIG welding, when the welding current was set to 115 A and the welding speed was 180 mm/min, the welding seam was well-formed and no welding defects appeared.

(2) The temperature field of dissimilar magnesium alloy AZ61-AM60 under TIG welding has been simulated. Since the thermal conductivity of magnesium alloy AM60 is larger than that of AZ61 at the same temperature, the thermal conductivity of AZ61 and AM60 is different. Thus, the temperature distribution of the molten pool shows a certain asymmetry on the AZ61 side and AM60 side.

(3) The simulation results show that, in the molten pool flow field on the AM60 side, the upper surface of the metal in molten pool moves from the center of the pool to the surroundings, then flows down along the boundary of the pool, finally flowing to the center of the molten pool when it is close to the center line of the welding seam. In addition, the movement direction of the AZ61 side is just symmetrical with that of the AM60 side, but the shape of the molten pool is asymmetrical.

(4) Comparing the simulation results of TIG welding and A-TIG welding, it can be found that, when A-TIG welding of dissimilar magnesium alloy AZ61-AM60 coated with activating flux is carried out (∂γ/∂T> 0), the surface of its molten pool is ingot-shaped. The penetration depth of the weld pool is large and the welding seam is narrow. Two grooves similar to the bite edge appear on the edge of the weld. During TIG welding (∂γ/∂T < 0), the welding surface is flat, the molten pool is shallow, and the welding seam is wider.

## Figures and Tables

**Table 1:** Alloy chemical composition (mass fraction/%).

Alloy	Al	Mn	Zn	Zr	Cu	Fe	Si	Mg
AZ61	5.8	0.18	1.0	–	0.003	–		balance
AM60	0.6	0.13	–	–	–	–		balance

**Table 2:** The thermophysical performance parameters of AZ61 [22].

Temperature (K)	Coefficient of Expansion (m·K^−1^)	Specific Heat (J·kg^−1^·K^−1^)	Thermal Conductivity (W·m^−1^·K^−^^1^)
373	24.4	1.13	73.27
473	26.5	1.21	79.55
573	31.2	1.26	79.55

**Table 3:** The thermophysical performance parameters of AM60 [23].

Temperature (°C)	Density (g·cm^−3^)	Young’s Modulus (Pa)	Poisson’s Ratio	Specific Heat (J·kg^−1^·K^−1^)	Thermal Conductivity (W·m^−1^·K^−1^)	Thermal Conductivity (W·m^−1^·K^−1^)
25	1.78683	4.62 × 10^10^	0.29214	1.01 × 10^3^	82.33476	2.49 × 10^−5^
100	1.77663	4.62 × 10^10^	0.29736	1.05 × 10^3^	87.01007	2.55 × 10^−5^
200	1.76237	4.62 × 10^10^	0.30434	1.10 × 10^3^	93.14288	2.64 × 10^−5^
300	1.74739	4.62 × 10^10^	0.31137	1.14 × 10^3^	99.21667	2.74 × 10^−5^
400	1.73174	3.56 × 10^10^	0.31843	1.18 × 10^3^	105.25875	2.83 × 10^−5^
500	1.71494	3.11 × 10^10^	0.32654	2.54 × 10^3^	111.30089	2.94 × 10^−5^
600	1.66912	2.06 × 10^9^	0.41178	6.64 × 10^3^	99.4966	4.09 × 10^−5^
700	1.61289	9.75 × 10^10^	0.49991	1.40 × 10^3^	79.93853	5.33 × 10^−5^
800	1.58666	4.09 × 10^10^	0.49996	1.40 × 10^3^	86.71718	5.43 × 10^−5^
